# An Implicit Registration Framework Integrating Kolmogorov–Arnold Networks with Velocity Regularization for Image-Guided Radiation Therapy

**DOI:** 10.3390/bioengineering12091005

**Published:** 2025-09-22

**Authors:** Pulin Sun, Chulong Zhang, Zhenyu Yang, Fang-Fang Yin, Manju Liu

**Affiliations:** 1 Medical Physics Graduate Program, Duke Kunshan University, Kunshan 215316, China; pulin.sun@duke.edu (P.S.); chulong.zhang@duke.edu (C.Z.); zhenyu.yang893@dukekunshan.edu.cn (Z.Y.); fangfang.yin@duke.edu (F.-F.Y.); 2 Jiangsu Provincial University Key (Construction) Laboratory for Smart Diagnosis and Treatment of Lung Cancer, Kunshan 215316, China

**Keywords:** CT–CBCT registration, implicit neural representation, Kolmogorov–Arnold Network, velocity field modeling, principal component analysis

## Abstract

In image-guided radiation therapy (IGRT), deformable image registration between computed tomography (CT) and cone beam computed tomography (CBCT) images remain challenging due to the computational cost of iterative algorithms and the data dependence of supervised deep learning methods. Implicit neural representation (INR) provides a promising alternative, but conventional multilayer perceptron (MLP) might struggle to efficiently represent complex, nonlinear deformations. This study introduces a novel INR-based registration framework that models the deformation as a continuous, time-varying velocity field, parameterized by a Kolmogorov–Arnold Network (KAN) constructed using Jacobi polynomials. To our knowledge, this is the first integration of KAN into medical image registration, establishing a new paradigm beyond standard MLP-based INR. For improved efficiency, the KAN estimates low-dimensional principal components of the velocity field, which are reconstructed via inverse principal component analysis and temporally integrated to derive the final deformation. This approach achieves a ~70% improvement in computational efficiency relative to direct velocity field modeling while ensuring smooth and topology-preserving transformations through velocity regularization. Evaluation on a publicly available pelvic CT–CBCT dataset demonstrates up to 6% improvement in registration accuracy over traditional iterative methods and ~3% over MLP-based INR baselines, indicating the potential of the proposed method as an efficient and generalizable alternative for deformable registration.

## 1. Introduction

Image registration transfers geometric structural information from one image to another and is indispensable in image-guided radiotherapy (IGRT). During the planning phase, clinicians delineate targets on a high-resolution kilovoltage planning CT (pCT), often with contrast enhancement and supplemented by MRI or PET when necessary [1]. It is well established that anatomical variations may occur during treatment due to organ filling, tumor shrinkage, or other factors. To account for these daily changes, on-board kilovoltage cone beam computed tomography (CBCT) is widely employed for real-time patient localization owing to its rapid acquisition and low radiation dose [2,3]. However, the utility of CBCT is limited by inherently low soft tissue contrast and projection artifacts. To compensate for these limitations, deformable image registration (DIR) between the reference pCT and daily CBCT has become an essential step in clinical workflows [4]. Based on appropriate deformation vector fields (DVFs), DIR integrates the accurate electron density from pCT with the real-time anatomy from CBCT, generating a predictive CT. Indeed, accurate deformable registration between these two modalities directly influences the precision of treatment planning and subsequent therapeutic outcomes.

Volumetric image registration remains challenging, particularly in CT–CBCT scenarios, due to multi-modal intensity discrepancies and complex anatomical deformations. To address this, various computational strategies have been developed, generally classified into two categories: traditional iterative methods and deep learning (DL)-based approaches. Iterative methods can be further divided into displacement-field-based and velocity-field-based (fluid) optimization. Displacement-based methods, such as Demons [5], local affine models [6], Elastix [7], and free-form deformation with B-splines [8], solve registration by directly optimizing the displacement vector field. While these methods are widely used, they often suffer from slow convergence, sensitivity to local minima, and the need for complex parameter tuning. Velocity-field-based methods, also known as fluid registration, model the transformation as a dynamic flow from the source image to the target image. By estimating a reasonable velocity field and integrating it, the deformation field between two images can be obtained, thereby achieving image registration. Due to incorporating temporal dynamics into the deformation process, these methods ensure reversibility and smoothness, and typically reduce topological folding rates [9]. Classic velocity-field-based frameworks include Large Deformation Diffeomorphic Metric Mapping (LDDMM) [10] and velocity Stationary Velocity Field (vSVF) [11]. vSVF assumes a time-invariant velocity, aiming to identify the initial momentum. In contrast, LDDMM treats the velocity as a function of position and time, optimizing for image similarity to determine the optimal initial momentum.

Over the past decade, deep learning has profoundly advanced biomedical image acquisition, processing, and interpretation by enabling hierarchical and transferable feature learning across modalities and anatomical regions. In musculoskeletal radiology, transfer learning has improved the detection of subtle knee MRI abnormalities despite limited annotations [12]; meanwhile, in stem cell biology, feature fusion frameworks have supported reliable classification of complex cellular phenotypes from heterogeneous photomicrographs [13]. These cross-domain successes underscore the importance of handling domain variability, which is particularly critical in medical image registration where anatomical diversity and protocol inconsistency often limit traditional algorithms. Unsupervised frameworks such as VoxelMorph [14] use convolutional neural networks to directly predict deformation fields by minimizing image dissimilarity with spatial regularization, achieving real-time inference and reducing computational cost compared with iterative methods. Extensions such as TransMorph [15] introduce transformers to capture global context, and DiffuseMorph [16] applies conditional diffusion models for flexible deformation representation. Despite promising results, CNN-based methods require large, diverse datasets and often generalize poorly to out-of-distribution inputs, while high memory demands typically necessitate image downsampling that compromises geometric fidelity. Although approaches like SynthMorph [17] mitigate data scarcity via synthetic training pairs, performance can degrade sharply when real testing data diverge from the synthetic priors, limiting clinical applicability in data-constrained or protocol-diverse environments.

In continuous physical modeling tasks such as medical image registration, implicit neural representation (INR) has emerged as a promising paradigm due to its continuous resolution and differentiability. By leveraging neural networks, INR approaches achieve adaptive nonlinear fitting and fundamentally overcome the representational limitations of traditional methods caused by sparse control points. Moreover, INR-based registration retains the characteristics of conventional per-case optimization, allowing the model to be trained from scratch for each image without requiring large training datasets. Several studies have demonstrated that INR approaches provide superior accuracy, and optimization stability in CT–CBCT registration tasks [18,19]. Notably, a key advantage of INR approaches is that they are defined in continuous coordinate space, enabling direct registration of images with different spatial resolutions without resampling or size normalization, thereby overcoming the reliance of conventional convolutional networks on uniform voxel size or resolution [20].

However, traditional INR implementations based on multilayer perceptron (MLP) face notable challenges when modeling high-dimensional velocity or deformation fields. A major limitation stems from the spectral bias inherent in MLP, which predisposes them to favor low-frequency components [21]. As a result, they struggle to accurately capture high-frequency anatomical structures and local deformations. To mitigate spectral bias, several advanced INR architectures have been proposed. For example, the sinusoidal representation network (SIREN) introduces periodic activation functions to enhance the representation of high-frequency components [22], while Fourier Feature Mapping augments input coordinates with high-frequency embeddings, effectively extending the bandwidth of the learned function [23]. These techniques improve the modeling of global high-frequency patterns, but they rely on a fixed spectral encoding determined before training, lacking the ability to locally adapt the frequency content according to spatially varying deformation complexity. Therefore, when applied to medical image registration, they may lack efficiency in representing the complexity of spatial variations. Recently, the Kolmogorov–Arnold Network (KAN) has been proposed as a promising alternative. Unlike conventional MLP that use fixed nonlinear activation functions, the KAN employs one-dimensional learnable parametric basis functions (e.g., B-splines) to construct nonlinear mappings through additive compositions. This architecture allows the KAN to perform adaptive spectral modeling [24], enabling better representation of high-frequency and localized deformation features.

In addition, implicit neural representations (INRs) that directly regress physical quantities such as velocity fields or signed distance functions also face serious computational and stability limitations. They often require iterative solutions of partial differential equations to optimize implicit fields, resulting in inference times longer than explicit methods [25,26], potentially exceeding clinically acceptable thresholds. More critically, their robustness remains in question, especially under dynamic or noise-perturbed conditions. In non-periodic dynamic CT reconstruction, INR approaches tend to produce anatomically implausible deformations when regressing DVF directly [27], often yielding overly complex local distortions—such as artifacts from rapid cardiac motion—without enforcing the physical continuity of biological tissues.

In this study, we propose a novel instance-optimization framework for CT–CBCT registration, which integrates the implicit representation capabilities of Kolmogorov–Arnold Network (KAN) with velocity-based regularization to enhance both the efficiency and robustness of medical image registration. The main contributions of this work are as follows:

1. We propose the first KAN-based implicit neural representation of the velocity field for CT–CBCT diffeomorphism registration.
2. The KAN network is use to encode velocity information into a compact representation, followed by inverse principal component analysis reconstruction, significantly improving registration efficiency.
3. The proposed approach is validated on a paired CT–CBCT public dataset from 19 pelvic patients, excellent registration accuracy was achieved.

## 2. Method

### 2.1. Method Overview

**Figure 1:**
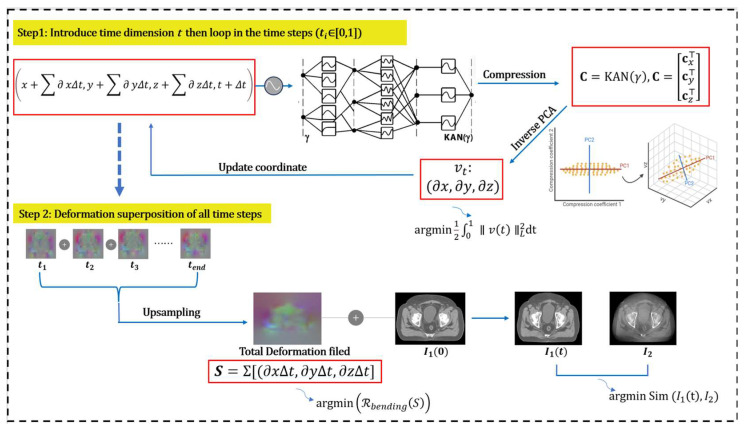
Illustration of the registration workflow. At each step, the time, *t*, and corresponding spatial coordinates (x,y,z,t) are used as inputs to predict the instantaneous velocity. This velocity is then used to compute the displacement increment. The updated position and the subsequent time step are then fed back into the KAN network to predict the next velocity, iteratively constructing the full deformation trajectory.

We propose a novel image registration framework that integrates three crucial components for efficient and accurate registration, as illustrated in Figure 1: a time–space-correlated velocity field; a Kolmogorov–Arnold Network (KAN) as an implicit function; an inverse PCA to reconstruct the global velocity field.

The deformation is parameterized as a flow field evolving over time. We considered the deformation, S, as the trajectory of fluid moving within a velocity field, governed by the ordinary differential equation in Equation (1). The time-dependent velocity field, v, is assumed to be sufficiently smooth to guarantee the existence of a unique, non-intersecting solution trajectory. To solve this initial value problem, we adopt a numerical integration approach using the forward Euler method over uniform time steps. The overall deformation is obtained by composing the incremental transformations across these time steps (as shown in Figure 1).

(1) $$\partial_{t}S\left(x,t\right)=v(t,S\left(x,t\right)),\;S(x,0)=x.$$

Our framework uniquely employs inverse principal component analysis to decompress high-dimensional velocity fields. Specifically, the spatiotemporal coordinates $\left(x,y,z,t\right)$ are first mapped to a low-dimensional latent space by the KAN, yielding the corresponding compression coefficients, c. Then, these coefficients are linearly combined with the PCA basis vectors, $\mu_{k}$, to reconstruct the velocity field in the original space.

### 2.2. Deformation Based on Implicit Representation

Position encoding module maps four-dimensional input coordinates to a higher-dimensional embedding, $\Gamma :R^{4}\to R^{d}\;(d⩾4).$ Specifically, it projects the input onto multi-frequency Fourier bases, making it easier for the KAN to capture fine-grained structural differences and local nonlinear deformation:

(2) $$\gamma =\left.\left.\Gamma (x,y,z,t)=\left[\begin{array}{c} \sin(b_{1}\pi x),\cos(b_{1}\pi x),\ldots ,\sin(b_{L}\pi x),\cos(b_{L}\pi x),\\ \sin(b_{1}\pi y),\cos(b_{1}\pi y),\ldots ,\sin(b_{L}\pi y),\cos(b_{L}\pi y),\\ \sin(b_{1}\pi z),\cos(b_{1}\pi z),\ldots ,\sin(b_{L}\pi z),\cos(b_{L}\pi z),\\ \sin(b_{1}\pi t),\cos(b_{1}\pi t),\ldots ,\sin(b_{L}\pi t),\cos(b_{L}\pi t) \end{array}\right.\right]\;\right.$$

where $b_{i}=i+1$ defines the linear frequency basis; this encoding maps 4D spatiotemporal coordinates to a d=4×2L dimensional space.

The encoded high-dimensional coordinates, γ, are inputted into the KAN network to estimate the instantaneous velocity vector, f(γ). The corresponding displacement for each time step is then accumulated. The coordinates are iteratively updated, and the cumulative displacement across all time steps yield the final displacement field. The whole process is formulated as:

(3) $$V_{t}=f(\gamma )=f(\Gamma \left(x,y,z,t\right))=\left(\partial x,\partial y,\partial z\right)$$

(4) $$\&(\Delta x,\Delta y,\Delta z)=V_{t}\times \delta t=(\partial x\Delta t,\partial y\Delta t,\partial z\Delta t)$$

(5) $$X_{t}=\Gamma \left(x+\Sigma \partial x\Delta t,y+\Sigma \partial y\Delta t,z+\partial z\Delta t,t+\Delta t\right)$$

(6) $$S=\Sigma \left[\left(\partial x\Delta t,\partial y\Delta t,\partial z\Delta t\right)\right]=\Sigma \;V_{t}\times \delta t\;$$

The optimization objective function comprises a similarity metric and two physical regularizers (as shown in Figure 1). A bending energy term and an $l^{2}$-norm regularize the displacement and velocity fields, respectively. The loss function is expressed as:

(7) $$L=arg\underset{S}{min}\left(Sim\left(I_{1}\circ S,I_{2}\right)+R_{bending}\left(S\right)+\frac{1}{n}\sum_{t=1}^{n}∥v_{t}{∥}_{L}^{2}\right)\;$$

It can be further derived that

(8) $$S=\sum_{t=1}^{n}f\left(\gamma \right)\times \delta t\;$$

(9) $$\begin{array}{cc} L= & \underset{S}{argmin}(\mathrm{Sim}(I_{1}\circ (\sum_{t=1}^{n}f(\gamma )\delta t),I_{2})\\ & +\frac{1}{n}\sum_{t=1}^{n}∥f\left(\gamma \right){∥}_{L}^{2}\;\\ & +R_{bending}\left(\sum_{t=1}^{n}f\left(\gamma \right)\delta t\right)) \end{array}$$

where n is the total number of time steps. In the *Sim* term, the operators **◦** and Σ denote the warping operation and the summation over all time steps, respectively.

The parameters of the KAN network are iteratively refined via gradient descent. This optimization procedure is repeated until a stopping criterion is met, ultimately producing the resultant deformation field, *S*.

### 2.3. KAN Network Modeling

The proposed methodology is centered on the Kolmogorov–Arnold Network, a framework that fundamentally diverges from the conventional multilayer perceptron. In this architecture, each scalar weight is replaced by a learnable, one-dimensional edge function, ϕ(x). This function is explicitly decomposed into two components: a base function for global trends and a polynomial series for local refinement. This is expressed as follows:

(10) $$\phi \left(x\right)=c_{base}\psi_{base}\left(x\right)+\sum_{n=0}^{5}c_{n}J_{n}^{\alpha ,\beta }\left(x\right)$$

Here, $\psi_{base}(x)$ is the fixed base function SiLU, while $J_{n}^{\alpha ,\beta }\left(x\right)$ are the 5th-order Jacobi basis functions. The coefficients $c_{base}$ and $c_{n}$ are learnable. These edge-centric functions are then aggregated at each neuron, where the activation $y_{j}$ is the simple sum of all incoming signals transformed by their respective edge functions:

(11) $$y_{j}=\sum_{i}\phi_{i,j}\left(x_{i}\right)$$

The power of this architecture stems from the expressiveness of the Jacobi basis. We leverage their orthogonality over the interval $\left[-1,1\right]$, with respect to a weight function $w^{\left(\alpha ,\beta \right)}\left(x\right)={\left(1-x\right)}^{\alpha }{\left(1+x\right)}^{\beta }$, formally expressed as:

(12) $$\int_{-1}^{1}J_{m}^{\alpha ,\beta }\left(x\right)J_{n}^{\alpha ,\beta }\left(x\right)w^{\left(\alpha ,\beta \right)}\left(x\right)dx=\delta_{mn}\;$$

where $\delta_{mn}$ is the Kronecker delta. This orthogonality guarantees that each basis term encodes a distinct frequency component, reducing parameter redundancy and cross-interference. Consequently, the parameterization remains numerically stable, enabling the network to optimize low- and high-frequency modes independently.

The network architecture is structured hierarchically. Following processing of 4D spatiotemporal coordinates through the positional encoding layer, the resulting feature vector propagates sequentially through a cascade of Jacobi–KAN layers. The final compressed coefficient matrix, $C\in R^{3\times D}$, is generated directly as the linear activation output of the terminal KAN layer, formally expressed as:

(13) $${\;C}_{k}=\sum_{j}\phi_{j,\mathrm{out}}\left(z_{j}\right)=\sum_{j}\left[c_{\mathrm{base},jk}\cdot \mathrm{SiLU}\left(z_{j}\right)+\sum_{n=0}^{5}c_{n,jk}J_{n}^{\left(\alpha ,\beta \right)}\left(z_{j}\right)\right]\;$$

where $z_{j}$ denotes the activation vector from the terminal hidden layer, $\phi_{j,\mathrm{out}}$ represents the edge-wise activation function in the output layer, and k={1,2,3} corresponds to the three dimensions of the displacement field.

Within a single forward pass, the model employs the same set of KAN parameters, $\Theta_{\mathrm{KAN}}$, to compute the velocity field at any given time step. This principle of time-invariant parameterization is formally expressed as:

(14) $$\Theta_{KAN}\left(t_{1}\right)=\Theta_{KAN}\left(t_{2}\right)\;\forall t_{1},t_{2}\in \left[0,T\right]\;$$

This design improves parameter efficiency by employing a shared parameter set across all time steps, thereby reducing model complexity and overfitting risk. Moreover, by modeling deformation as a continuous function of time, it enforces temporal Lipschitz continuity of the velocity field, ensuring a physically plausible transformation.

(15) $$∥V\left(x,y,z,t+\Delta t\right)-V\left(x,y,z,t\right)∥\leq L∥\Delta t∥$$

### 2.4. Inverse PCA for Reconstructing the Velocity Field

#### 2.4.1. Motivation

Predicting compressed coefficients rather than directly regressing high-dimensional velocity fields helps mitigate the challenges associated with dense output modeling. Neural architectures often struggle with the computational and representational demands of high-resolution predictions, and increasing resolution (e.g., to 128 × 128) can introduce greater model complexity and overfitting risk, without corresponding gains in accuracy, as observed in DCT-based masking frameworks [28]. Moreover, velocity fields are typically spatially sparse and dominated by low-frequency components. These characteristics make dimensionality reduction an effective strategy for balancing computational efficiency and reconstruction fidelity.

**Figure 2:**
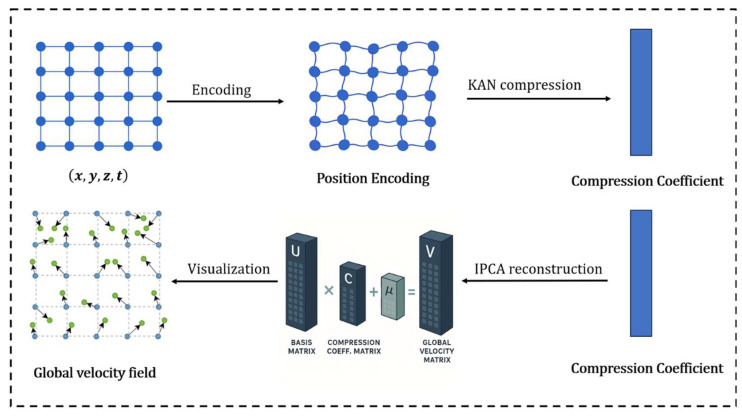
Schematic diagram of compressing and decompressing the velocity field. The full global velocity field is reconstructed by linearly combining the compression coefficient matrix, V, with the principal component basis, U, followed by the addition of the mean velocity field, μ. In the global velocity field, blue dots denote initial positions, while the green dots mark their locations after transformation.

We integrate an inverse principal component analysis decoder into the pipeline to reconstruct the velocity field from predicted low-dimensional coefficients. Prior studies support this design, Shu et al. modeled temporal deformation modes in 4D-CBCT using PCA, reducing both computational cost and variability [29], while Alexander et al. demonstrated that PCA-based reconstruction maintains anatomical plausibility and generalizes well in head-and-neck registration tasks [30]. Leveraging these insights, our method achieves an effective balance between computational efficiency and anatomical fidelity. The overall framework is shown in Figure 2.

#### 2.4.2. Mathematical Derivation

Once the KAN network predicts the compression coefficient, *C*, the inverse PCA can reconstruct the velocity field for the entire grid, which is essentially a linear decoding process. The matrix of compression coefficients is as follows:

(16) $$C=\left[\begin{array}{cccc} c_{x}^{\left(1\right)} & c_{x}^{\left(2\right)} & \cdots & c_{x}^{\left(k\right)}\\ c_{y}^{\left(1\right)} & c_{y}^{\left(2\right)} & \cdots & c_{y}^{\left(k\right)}\\ c_{z}^{\left(1\right)} & c_{z}^{\left(2\right)} & \cdots & c_{z}^{\left(k\right)} \end{array}\right]\;$$

where *K* is the compression dimension, derived based on the original number of grids. For example, when the number of grids, *N*, of the velocity field is 16 × 24 × 24 and the compression ratio is 0.1, *K* = 921.

Furthermore, multiplying the compression coefficient matrix by the base matrix, U, and adding the average offset, μ, can restore the complete velocity field, thus achieving the transformation from low dimensional compressed representation to high-dimensional velocity field.

(17) V=CU+μ

To be more specific,

$$\left[\begin{array}{ccc} v_{x}^{\left(1\right)} & \cdots & v_{x}^{\left(N\right)}\\ v_{y}^{\left(1\right)} & \cdots & v_{y}^{\left(N\right)}\\ v_{z}^{\left(1\right)} & \cdots & v_{z}^{\left(N\right)} \end{array}\right]=\underset{C}{\underbrace{\left[\begin{array}{cccc} c_{x}^{\left(1\right)} & c_{x}^{\left(2\right)} & \cdots & c_{x}^{\left(k\right)}\\ c_{y}^{\left(1\right)} & c_{y}^{\left(2\right)} & \cdots & c_{y}^{\left(k\right)}\\ c_{z}^{\left(1\right)} & c_{z}^{\left(2\right)} & \cdots & c_{z}^{\left(k\right)} \end{array}\right]}}\cdot \underset{U}{\underbrace{\left[\begin{array}{ccc} \phi_{1}^{\left(1\right)} & \cdots & \phi_{1}^{\left(N\right)}\\ \vdots & \ddots & \vdots \\ \phi_{k}^{\left(1\right)} & \cdots & \phi_{k}^{\left(N\right)} \end{array}\right]}}+\underset{\mu }{\underbrace{\left[\begin{array}{ccc} \mu_{x}^{\left(1\right)} & \cdots & \mu_{x}^{\left(N\right)}\\ \mu_{y}^{\left(1\right)} & \cdots & \mu_{y}^{\left(N\right)}\\ \mu_{z}^{\left(1\right)} & \cdots & \mu_{z}^{\left(N\right)} \end{array}\right]}}$$

Unlike traditional singular value decomposition that derives basis functions from the data covariance matrix, our method treats both U and μ as learnable parameters, jointly optimized with the KAN network via backpropagation during training. We assert that deformation follows a Gaussian distribution, where initial randomness is introduced to allow each basis vector to explore distinct motion directions. During training, gradient contributions are adaptively weighted at each iteration to balance image similarity and motion smoothness, guiding *U* to converge toward the principal motion subspace.

(18) $$\left\{\begin{array}{c} \;\;\;\;U^{\left(0\right)}=N(0,0.01)\in R^{k\times N}\\ \;\;\;\;\mu^{\left(0\right)}=0\in R^{N}\\ \;\;\;\;\left[\begin{array}{c} U^{\left(t+1\right)}\\ \mu^{\left(t+1\right)} \end{array}\right]=\left[\begin{array}{c} U^{\left(t\right)}\\ \mu^{\left(t\right)} \end{array}\right]-\eta \nabla L \end{array}\;\right.$$

### 2.5. Coarse-to-Fine Strategy

To further mitigate the computational burden, a coarse-to-fine strategy was adopted. Specifically, the velocity field is first predicted on a low-resolution control grid (e.g., 16 × 24 × 24), which significantly reduces the parameter space compared to voxel-wise prediction. The displacement field is then reconstructed through trilinear interpolation and upsampled to the target image resolution (e.g., 96 × 128 × 128). During this process, displacement amplitudes are proportionally scaled to maintain physical consistency across resolutions. This hierarchical scheme not only reduces memory consumption and accelerates training but also enforces spatial smoothness in the resulting deformation. The dense displacement field is subsequently applied to warp the moving image via standard spatial transformation.

## 3. Experiment

### 3.1. Dataset

The publicly available dataset used in this study is the Pelvic Reference Dataset (PRD) [31], sourced from the Cancer Imaging Archive (https://www.cancerimagingarchive.net, accessed on 18 March 2025). PRD includes paired of pelvic CT and CBCT in 58 subjects. Cone beam CT images were acquired within one week following the corresponding planning CT scans. Prior to inclusion, all cases were independently reviewed by two certified radiation oncologists to assess their suitability for image registration analysis. The screening process involved a standardized checklist that evaluated four key criteria: (1) anatomical completeness, (2) image quality (especially artifact severity), (3) consistency in patient positioning, and (4) adequate field-of-view (FOV) coverage of the region of interest. Discrepancies between reviewers were resolved through consensus discussion. Following this review process, a total of 19 cases (15 male, 4 female) were selected.

### 3.2. Image Preprocessing

**Figure 3:**
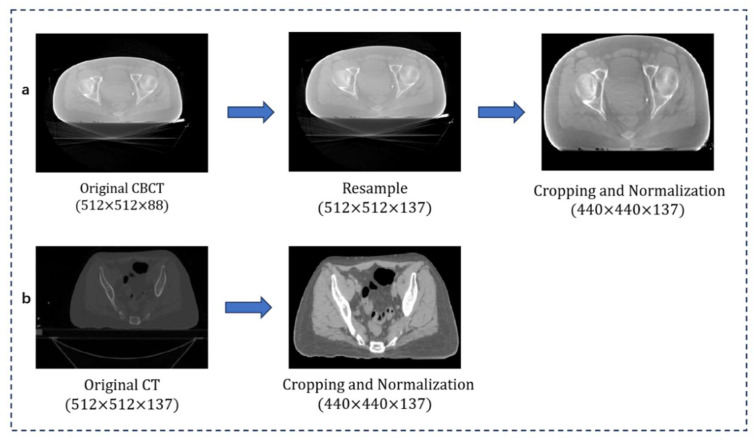
Image preprocessing. (**a**) Resampling, cropping, and intensity normalization of CBCT. (**b**) CT cropping and intensity normalization.

The CT and CBCT volumes were acquired with an in-plane resolution of 512 × 512 pixels, corresponding to a pixel size of 1.00 × 1.00 mm^2^. The slice thickness was 3.00 mm for both modalities. Each CBCT volume consisted of 88 slices, while the number of CT slices varied across patients, exceeding 100. To ensure minimal geometric distortion, all CBCT volumes were resampled to match the spatial dimensions of the corresponding CT scans. Each CT–CBCT pair was individually cropped based on its anatomical characteristics, as the optimal bounding region varied across cases. This process focused on excluding unrelated structures such as the treatment couch. To reduce intensity discrepancies and enhance contrast, CT and CBCT images were intensity-normalized. Specifically, CT images were clipped to [−300, 250] HU and CBCT images to [−600, 200] HU, followed by linear scaling to the [0, 1] range. The overall framework is shown in Figure 3.

### 3.3. Organ Contour Delineation

For each patient, four anatomical structures were delineated on both the planning CT (pCT) and CBCT: femur, hip bone, bladder, and rectum. The femur and hip bones were initially segmented using TotalSegmentator (version 2.5.0) [32] and then manually refined to correct inaccuracies. In contrast, the bladder and rectum, due to their high inter-patient variability and poorly defined boundaries, were manually contoured by experienced radiologists. To reduce annotation bias and ensure consistency, all contours were independently reviewed by a second expert, and only those that met consensus quality criteria were included in the registration experiments.

### 3.4. Evaluation

In this study, we evaluated the proposed registration framework from three perspectives: (1) registration efficiency, assessed by convergence trend; (2) using the standard image similarity measure, the Dice Similarity Coefficient (DSC), and 95% Hausdorff surface distance (${HD}_{95})$ to evaluate registration accuracy and compare it with the existing advanced methods; (3) the quality of the deformation field was evaluated based on Jacobian determinant analysis. This multi-dimensional evaluation was designed to verify the soundness of the method in clinical image registration tasks.

#### 3.4.1. Efficiency of Inverse PCA

**Figure 4:**
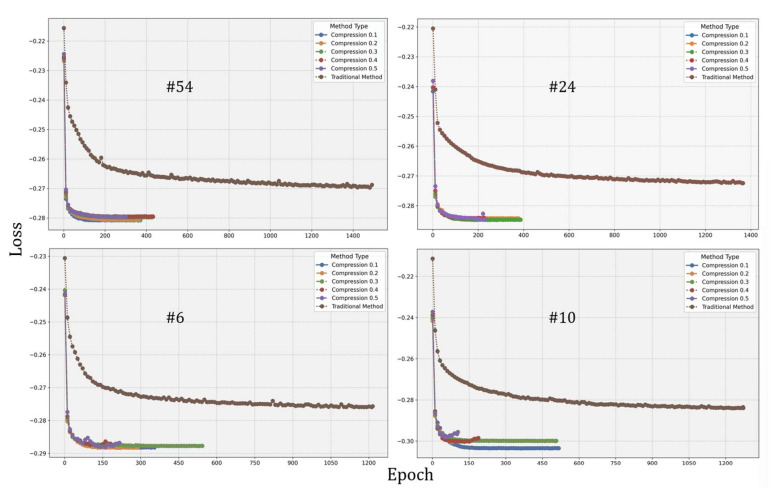
Convergence comparison curves of four representative examples (# 54, # 24, # 6, # 10). The horizontal axis is epoch, and the loss value is used as the vertical axis.

To evaluate the efficiency gains from introducing inverse PCA, convergence behaviors were compared under five compression ratios against direct velocity field prediction. The specific results are shown in Figure 4. While the conventional INR method demonstrated stable convergence across all cases, it required more training epochs and converged more slowly. In contrast, the inverse PCA-based approach achieved significantly faster convergence with fewer epochs and reached lower loss values, indicating superior performance. Rapid early-stage convergence was observed across all compression ratios. However, at higher ratios (e.g., 0.4 and 0.5), training instability includes oscillation and premature stagnation (in the case of #6 and #10). This may result from redundant information or increased optimization difficulty associated with higher-dimensional latent representations.

Overall, the proposed method significantly accelerates convergence and maintains registration accuracy at appropriate compression levels (e.g., ratios of 0.1–0.2), but excessive retention of compressed features (e.g., ratios of 0.4–0.5) may introduce instability, highlighting the need for careful tuning of compression parameters.

#### 3.4.2. Accuracy Evaluation of Registration

The Dice Similarity Coefficient (DSC) was used to evaluate volumetric overlap, while the 95th percentile Hausdorff Distance (HD_95_) was applied to assess boundary agreement for the femur, hip, bladder, and rectum. The Dice Similarity Coefficient (DSC) [33] quantifies the spatial overlap between predicted and ground-truth segmentations and is widely used to evaluate segmentation accuracy. The Hausdorff Distance (HD) [34] is defined as the maximum Euclidean distance from each point on the target contour to its nearest neighbor on the deformed contour, calculated on a per-slice and averaged across all slices. To mitigate the influence of outlier points, the HD_95_ was used instead, representing the 95th percentile of the distribution of all surface-to-surface distances.

**Figure 5:**
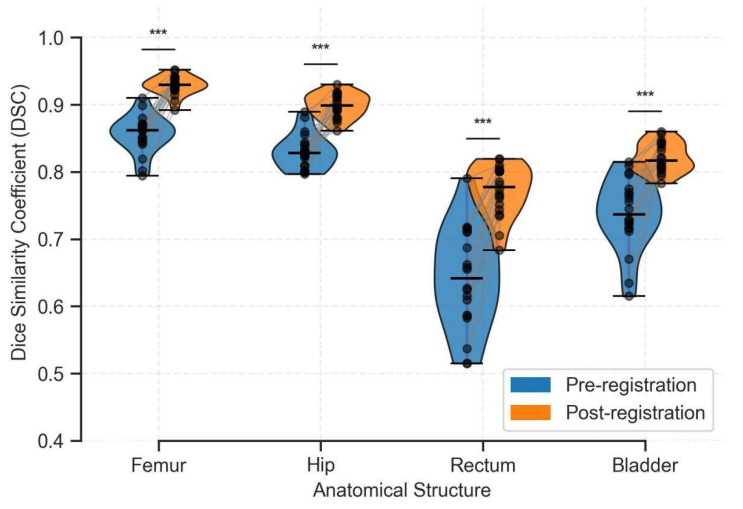
Comparison of DSC results of the entire test set before and after registration of four segmentation labels. The blue violin image represents unregistered, while the orange violin represents the result after deformation registration. The asterisk symbol on each organ represents statistically significant differences (*p* < 0.001).

Figure 5 shows violin plots of DSC values. After registration, DSC distributions changed significantly (*p* < 0.001) and exhibited reduced inter-patient variability. Even the femur and hip, initially well-aligned, showed certain improvements. Notably, the bladder and rectum, despite high anatomical variability, demonstrated substantial improvements, with median DSC rising from 0.64 to 0.79 for the rectum and from 0.74 to 0.83 for the bladder.

Table 1 presents a quantitative comparison between the proposed method and baseline approaches. Given the limited sample size, deep-learning-based methods encounter challenges in training and generalization. Thus, we focus on comparisons with iterative optimization-based approaches. Our method demonstrates superior performance, achieving an improvement of over 6% in the average Dice Similarity Coefficient (DSC) compared to iterative-based approaches. The average 95th percentile Hausdorff Distance (HD_95_) was reduced from 7.01 mm to 5.09 mm. Two key findings emerge. First, INR-based methods consistently outperform conventional iterative approaches. Through ablation experiments, it can be found that the improvement of using KAN instead of MLP is limited, but incorporating with velocity field can generate obvious gains, emphasizing its key role in improving registration accuracy.

**Figure 6:**
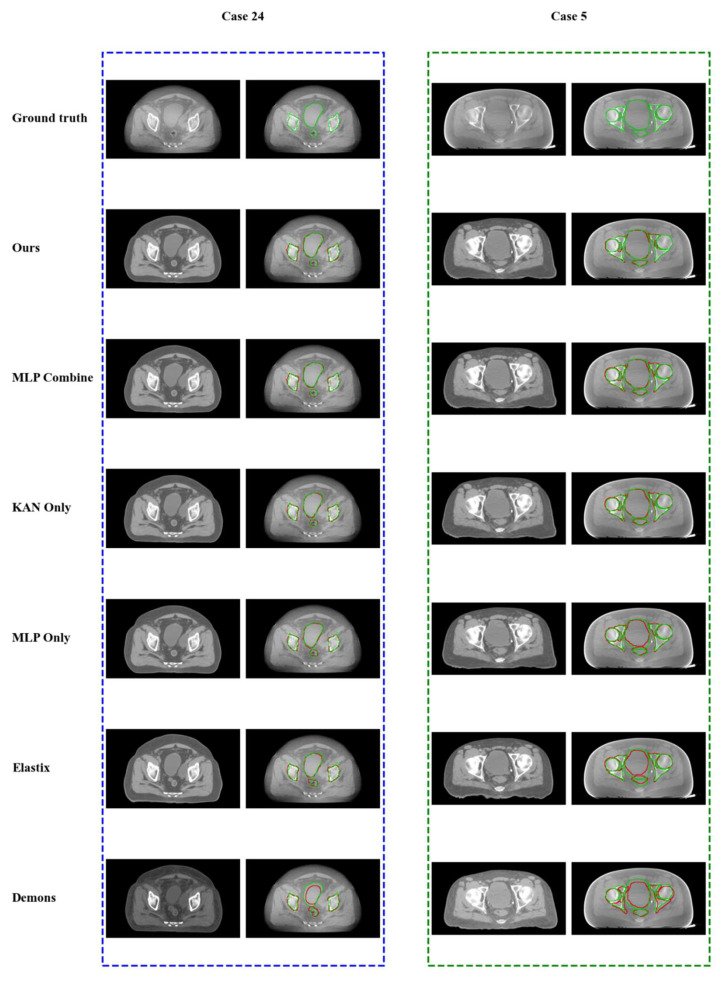
Visual comparison of registration results for two representative patient cases (24 and 5) using five different methods. For each case, the target CBCT and real contour (green) are displayed on the first row. The remaining rows show the moved CT and predicted contours (red) generated by different methods. Regions of overlap appear green, while mismatches are highlighted in red.

**Figure 7:**
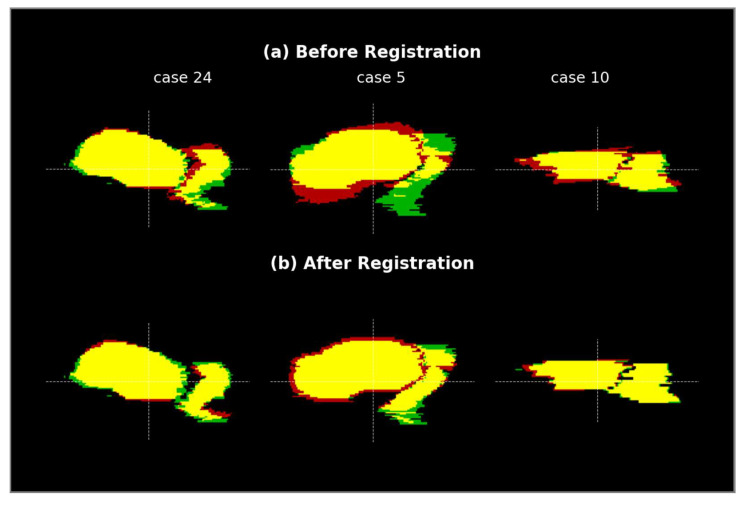
Organ alignment before and after registration in sagittal view. Red denotes the moving CT region, green indicates the fixed CBCT, and yellow highlights their overlapping area.

Figure 6 provides a visual comparison of segmentation results. In these images, the green contours represent the ground truth, while the red contours denote the predicted segmentations. Overlapping regions are also displayed in green. As shown, the proposed method produces contours that exhibit closer alignment with the ground truth. Specifically, Case 24 exhibited relatively small anatomical deformation, and all five methods achieved satisfactory registration results. Notable discrepancies were observed in the rectal region for the Elastix and Demons methods. In contrast, Case 5 involved larger deformations, for which our method produced more accurate results. It is worth noting that for Case 5, the limited field of view (FOV) in the CBCT scan adversely affected the accuracy of peripheral contour prediction. Furthermore, sagittal views in Figure 7 highlight the improved alignment of the bladder and rectum in these three patients following registration with our method.

#### 3.4.3. Quality of Deformation Field

The Jacobian determinant is a widely used metric for assessing the anatomical plausibility and physical regularity of deformation fields in image registration. It quantifies local volumetric changes induced by a spatial transformation. For a 3D displacement field, $u\left(x\right)$, the transformation is defined as $\phi \left(x\right)=x+u\left(x\right)$, and the Jacobian matrix is given by:

(19) $$J\left(x\right)=\nabla \phi \left(x\right)=I+\nabla u\left(x\right)$$

where I is the identity matrix and $\nabla u\left(x\right)$ denotes the gradient of the displacement field. The Jacobian determinant $det\left(J\right)$ reflects the local volume change. Critically, $det\left(J\right)<0$ denotes folding or non-injective mapping, which violates topology preservation and is considered anatomically implausible.

**Figure 8:**
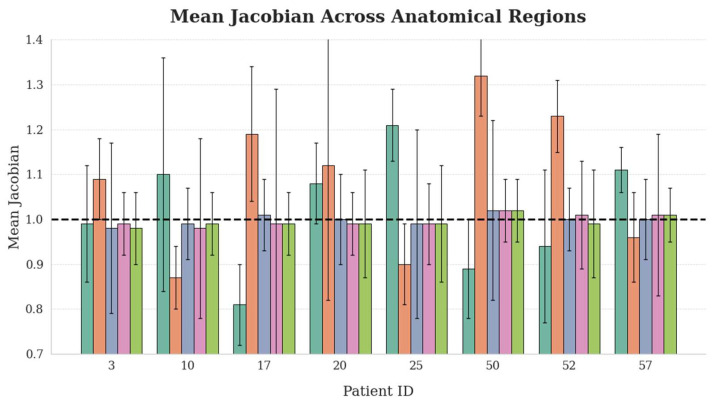
Analysis of mean Jacobian determinants across anatomical regions and patients.

In this study, Jacobian determinant maps were computed using finite difference approximations. Global and regional distributions were evaluated using key metrics, including the percentage of negative values, the proportion of moderately deformed voxels (0.7 ≤ det(J) ≤ 1.3), and the basic statistics. As summarized in Figure 8 and Table 2, the results demonstrate that the proposed registration method generates more anatomically plausible and topologically consistent deformation fields.

Figure 8 shows the mean Jacobian determinants under the proposed method for several representative patients. Rigid structures (femur and hip) maintained mean Jacobian values near 1.0 with minimal variation (SD ≈ 0.01), indicating high local stability. In contrast, soft tissues such as the bladder and rectum exhibited greater variability (SD > 0.11) and deviations from 1.0, reflecting physiologically realistic deformation. As summarized in Table 2, the proposed method achieved statistically superior deformation field quality compared to traditional iterative and standard INR-based methods. All approaches preserved topology (minimum Jacobian > 0.16). However, ours yielded the most uniform volume changes, with the lowest global standard deviation. Notably, severe voxel compression (det(J) < 0.3) in the bladder was reduced, and over-expansion was effectively constrained. Spatial distribution analysis further confirmed smoother deformation transitions. Our method achieved the highest anatomically plausible deformation ratio, with moderate deformations accounting for 99.343% globally, and 90.007% and 86.133% in the bladder and rectum, respectively. These results demonstrate that the proposed framework provides topologically consistent, volumetrically stable, and anatomically accurate deformation fields for pelvic image registration.

Here, we present a summary of the Jacobian determinant analysis across different regions of interest for all methods. The table shows the mean, standard deviation, minimum, and maximum values of the Jacobian, as well as the percentage of voxels with moderate deformation.

## 4. Discussion

Accurate registration between planning CT (pCT) and daily CBCT scans is critical for target localization and dose delivery in image-guided radiation therapy (IGRT). Traditional registration techniques typically rely on iterative optimization algorithms or neural networks to predict the deformation vector field (DVF) between images, which can be computationally intensive and prone to being stuck in local optima. Diffeomorphic flow-based models are valued for their smooth, invertible transformations, yet their reliance on high-dimensional velocity fields introduces inefficiencies and overparameterization.

This study presents an instance-specific optimization framework for image registration, departing from conventional approaches that train general networks to predict deformation fields from image pairs. Instead, we construct a compact and dedicated neural representation for each case by directly optimizing a lightweight model. At the core of this design is an implicit parameterization of the velocity field using a Jacobi–KAN network, which captures a low-dimensional latent representation. Rather than operating on full-resolution velocity fields, the method encodes the deformation in a small number of trainable weights, greatly reducing model complexity and the risk of overfitting.

To reconstruct high-resolution deformation fields from a compact latent space, a learnable inverse PCA decoder is introduced, which can efficiently and smoothly recover velocity fields. This design fundamentally compresses the parameter space to be optimized. Moreover, the scheme of generating the final deformation field based on the integration of the velocity field ensures that the generated deformation path is continuous and physically real in time and space.

The proposed method achieves high performance on the challenging task of pelvic image registration. On average, it improves registration accuracy by 6% over traditional iterative methods and by approximately 3% as compared to the MLP-based INR method. Notably, the integration of KAN with an inverse PCA decoder enhances computational efficiency by ~70% without compromising accuracy. This improvement stems from replacing direct per-voxel velocity optimization with a compact, learnable latent representation.

An additional strength of our method lies in the quality of the resulting deformation fields. Jacobian determinant analysis confirms the absence of folding across the entire image domain, indicating that the deformation preserves anatomical topology without introducing unrealistic compression or tearing. This benefit arises from the combination of multi-step integration and the smoothness constraints imposed by the inverse PCA decoder, ensuring that the deformation is both physically plausible and anatomically coherent.

Nevertheless, the proposed method has several limitations. Due to the physiological variability of bladder and rectum, registration accuracy in these regions remains suboptimal. The Dice Similarity Coefficient for the bladder rarely exceeds 0.85, while values for the rectum seldom reach 0.80. Another limitation lies in the relatively small sample size used in this study, which restricts the generalizability of our evaluation. The method has not been tested on other common deformation patterns, such as respiratory-induced thoracic motion, limiting assessment of robustness. In addition, fair comparisons with deep-learning-based models were not conducted, as such models typically require large-scale datasets to demonstrate their strengths. Under the current data conditions, our instance-specific optimization approach is more naturally applicable, thereby future studies should include comparisons with learning-based methods.

Translating the proposed framework into clinical practice requires addressing key challenges in validation and workflow integration. Before clinical deployment, extensive validation on large-scale internal datasets is necessary to establish robust performance benchmarks. Comprehensive debugging and quality assurance (QA) protocols must be established, assessing not only geometric accuracy (such as target alignment) but also evaluating the physical rationality of the deformation field through Jacobian analysis and its dosimetric impact. To facilitate adoption, instance-specific optimized workflows should be encapsulated as modular software components that can be integrated into commercial treatment planning systems (TPSs), to receive image pairs from the TPS, perform rapid optimization, and return the DVF for plan evaluation.

To conclude, the proposed method leverages a velocity field framework that integrates KAN-based implicit representation with PCA dimensionality reduction, enabling the generation of smooth and anatomically consistent deformation fields with improved accuracy and efficiency. This offers a promising direction for medical image registration. Future work may incorporate explicit anatomical priors, such as boundary constraints derived from segmentation contours, to address challenges in highly deformable regions. Furthermore, the optimal PCA compression ratio, which balances registration accuracy against computational efficiency, currently requires manual selection. Proposing an adaptive or data-driven method for selecting this ratio on a case-by-case basis is a highly promising future research direction. Additionally, systematic validation on larger, more diverse datasets will be essential in enhancing clinical applicability.

## 5. Conclusions

We proposed a CT–CBCT registration framework based on a Kolmogorov–Arnold Network (KAN) with a neural network and velocity field modeling. Without a pretraining process, a dedicated deformation model is built and optimized in real time for each image pair, enhancing their individual adaptability. The velocity field was implicitly represented and then reconstructed via inverse PCA, to reduce parameter dimensionality and computational cost. A continuous and smooth displacement field was reconstructed through multi-step integration, demonstrating superior accuracy and robustness in the challenging task of pelvic registration.

## Figures and Tables

**Table 1:** Comparison of average DSC and HD_95_ results from multiple methods. The best value is shown in bold font, and the second best value is underlined.

Type	Method	Femur	Hip	Bladder	Rectum	Average
(a) DSC (unit: %)						
Iteration-based	Demons	87.89 ± 2.24	86.65 ± 2.91	74.11 ± 4.65	70.33 ± 5.79	79.75
	Elastix	88.09 ± 3.01	88.87 ± 2.77	75.13 ± 3.16	71.91 ± 3.65	81.00
INR-based	MLP only	90.91 ± 4.06	90.11 ± 2.65	79.44 ± 4.14	74.03 ± 4.17	83.62
	KAN only	91.18 ± 3.34	90.37 ± 2.30	80.15 ± 3.05	76.23 ± 3.81	84.48
	MLP combine	92.77 ± 2.88	90.45 ±2.35	81.88 ± 3.91	78.49 ± 3.52	85.90
	Ours	**9** **3.09 ± 2.15**	**9** **0.88 ± 2.63**	**8** **2.73 ± 4.74**	**79.42 ± 3.19**	**8** **6.53**
(b) HD_95_ (unit: mm)						
Iteration-based	Demons	4.35 ± 1.42	5.47 ± 1.21	8.96 ± 3.41	9.25 ± 3.01	7.01
	Elastix	4.15 ± 1.17	5.17 ± 1.36	8.59 ± 4.01	8.93 ± 3.26	6.71
INR-based	MLP only	3.40 ± 1.43	4.26 ± 0.97	7.01 ± 3.92	7.33 ± 3.98	5.50
	KAN only	3.32 ± 1.56	4.24 ± 1.14	6.94 ± 4.01	7.28 ± 3.83	5.45
	MLP combine	3.13 ± 1.16	4.16 ± 0.93	6.27 ± 3.03	**7.21 ± 3.15**	5.19
	Ours	**2.97 ± 1.03**	**4.06 ± 1.01**	**6.11 ± 3.14**	7.23 ± 2.79	**5.09**

**Table 2:** Analysis of mean Jacobian determinants across anatomical regions.

Method	Mean Jacobian	Std. Dev.	Min Value	Max Value	Moderate (%)
(a) Demons					
Bladder	0.946	0.119	0.240	2.460	85.612
Femur	0.993	0.017	0.560	1.590	99.619
Global	0.986	0.019	0.110	3.370	98.713
Hip	0.991	0.017	0.500	1.700	99.401
Rectum	1.122	0.131	0.290	2.510	80.334
(b) Elastix					
Bladder	0.951	0.120	0.270	2.410	88.308
Femur	0.995	0.018	0.580	1.590	99.770
Global	0.989	0.019	0.130	3.130	98.776
Hip	0.994	0.016	0.500	1.700	99.524
Rectum	1.113	0.121	0.290	2.450	81.051
(c) MLP only					
Bladder	0.976	0.115	0.310	2.200	88.540
Femur	0.998	0.012	0.580	1.550	99.839
Global	0.993	0.015	0.160	2.950	99.046
Hip	0.997	0.013	0.510	1.500	99.629
Rectum	1.080	0.118	0.350	2.280	82.397
(d) KAN only					
Bladder	0.976	0.115	0.320	2.170	89.214
Femur	0.998	0.012	0.580	1.550	99.844
Global	0.994	0.015	0.160	2.920	99.117
Hip	0.998	0.013	0.510	1.490	99.635
Rectum	1.080	0.116	0.350	2.260	83.481
(e) MLP combine					
Bladder	0.977	0.115	0.330	2.100	89.975
Femur	0.998	0.011	0.580	1.530	99.844
Global	0.994	0.014	0.160	2.890	99.227
Hip	0.998	0.012	0.50	1.480	99.644
Rectum	1.079	0.113	0.360	2.260	85.792
(f) Ours					
Bladder	0.978	0.113	0.330	2.060	90.007
Femur	0.998	0.012	0.590	1.530	99.859
Global	0.995	0.013	0.160	2.870	99.343
Hip	0.998	0.011	0.520	1.480	99.656
Rectum	1.078	0.112	0.370	2.250	86.133

## Data Availability

The dataset and images used for this study are publicly available on the Pelvic Reference Dataset (PRD) at https://www.cancerimagingarchive.net (accessed on 11 May 2025).

## Abbreviations

The following abbreviations are used in this manuscript:

CT	computed tomography
CBCT	cone beam computed tomography
KAN	Kolmogorov–Arnold Network
PCA	principal component analysis
pCT	planning computed tomography
IGRT	image-guided radiation therapy
INR	implicit neural representation
3D	three-dimensional
MLP	multilayer perceptron
DSC	Dice Similarity Coefficient
HD	Hausdorff Distance
